# Deubiquitinase USP35 regulates MDM4 degradation to promote endothelial ferroptosis and renal injury progression

**DOI:** 10.1038/s41420-026-03128-5

**Published:** 2026-05-25

**Authors:** Chenyang Han, Li Guo, Wenyan Li, Shasha Wu, Jian Sheng, Yahui Lv, Yi Yang, Ming Li, Zhiling Tang

**Affiliations:** 1 Department of Pharmacy, The Second Affiliated Hospital of Jiaxing University, Jiaxing City, Zhejiang China; 2 Department of Radiotherapy, The Second Affiliated Hospital of Jiaxing University, Jiaxing City, Zhejiang China; 3 Department of Urology, The Second Affiliated Hospital of Jiaxing University, Jiaxing City, Zhejiang China

**Keywords:** Chemical modification, Acute kidney injury

## Abstract

Cisplatin is a widely used chemotherapeutic drug for various types of malignant tumors, but its nephrotoxic side effects limit its clinical application. There have been numerous reports and studies on cisplatin-induced acute kidney injury (AKI). Through transcriptomic and single-cell analyses, we found that USP35 is highly expressed in AKI and is closely related to MDM4. We investigated the role and mechanism of USP35-mediated MDM4 ubiquitination in AKI using renal epithelial cells and animal models. In a kidney-specific USP35 knockout model, AKI was significantly improved, and endothelial ferroptosis was inhibited. Overexpression of USP35 can promote AKI and endothelial ferroptosis. USP35 induces the autophagic degradation of MDM4 following deubiquitination, which activates the P53 signaling pathway mediating endothelial ferroptosis. We propose that the USP35-MDM4-P53 signaling pathway plays an important role in AKI and indicates that USP35 is a potential therapeutic target for AKI.

## Introduction

Acute kidney injury (AKI) is a kidney disease characterized by a rapid decline in renal function [1, 2]. Cisplatin is a commonly used chemotherapeutic drug, and its clinical application is widespread. However, high doses or prolonged use of cisplatin can induce nephrotoxicity, leading to AKI [3–5]. This limits the use of cisplatin. Iron can participate in the Fenton reaction to produce hydroxyl radicals [6], which can cause non-selective damage to most biomacromolecules. When there is iron overload in cells, it leads to lipid peroxidation [7]. This form of regulated cell death, characterized by lipid peroxidation and dependent on iron regulation, is named ferroptosis [8]. Studies on AKI have found that iron overload can induce ferroptosis in renal tubular epithelial cells and is involved in the occurrence of AKI [9]. Ferroptosis inhibitors have shown renal protective effects in various AKI animal models, indicating that ferroptosis may play an important role in the occurrence and development of AKI and is a new avenue for AKI treatment [10, 11].

Deubiquitination, as a post-translational modification, plays a key role in regulating proteasomal degradation or the life processes of other substrate proteins [12]. An increasing number of studies have found that deubiquitinases play important roles in various diseases. In reports on renal injury [13, 14], JOSD2 can effectively prevent renal tubular injury and inflammation in AKI mice. SIRT7 is a potential substrate of JOSD2. JOSD2 removes the K63-linked ubiquitination of SIRT7 through its active site C24 and promotes p62-mediated autophagic degradation of SIRT7, thereby preventing the phosphorylation and nuclear translocation of p65 and reducing the inflammatory response of renal tubular epithelial cells [15]. OTUD1 binds to CDK9 and induces deubiquitination of CDK9, which in turn induces inflammatory responses and fibrosis in renal epithelial cells [16]. Through transcriptomic analysis, we found that USP35, a deubiquitinase, is highly expressed in acute kidney injury and is closely related to MDM4 and MDM2. USP35 is a member of the USP subfamily of DUBs, mainly formed by ZNF and UBA structures [17], and can function through deubiquitination of Lys63/48/27. The role of USP35 in AKI has not yet been reported.

We investigated the role of USP35 in cisplatin-induced AKI. The results showed that USP35 interacts with MDM4 through deubiquitination. MDM4 further binds to p62 and undergoes autophagic degradation. The degradation of MDM4 can further promote the activation of P53, leading to the occurrence of endothelial ferroptosis. The USP35-MDM4-P53 pathway is a novel regulatory signal in cisplatin-induced AKI. Our study indicates that USP35 plays an important role in AKI, promoting its progression, and is related to the activation of endothelial ferroptosis.

## Results

### Upregulation of USP35 in AKI

**Fig. 1:**
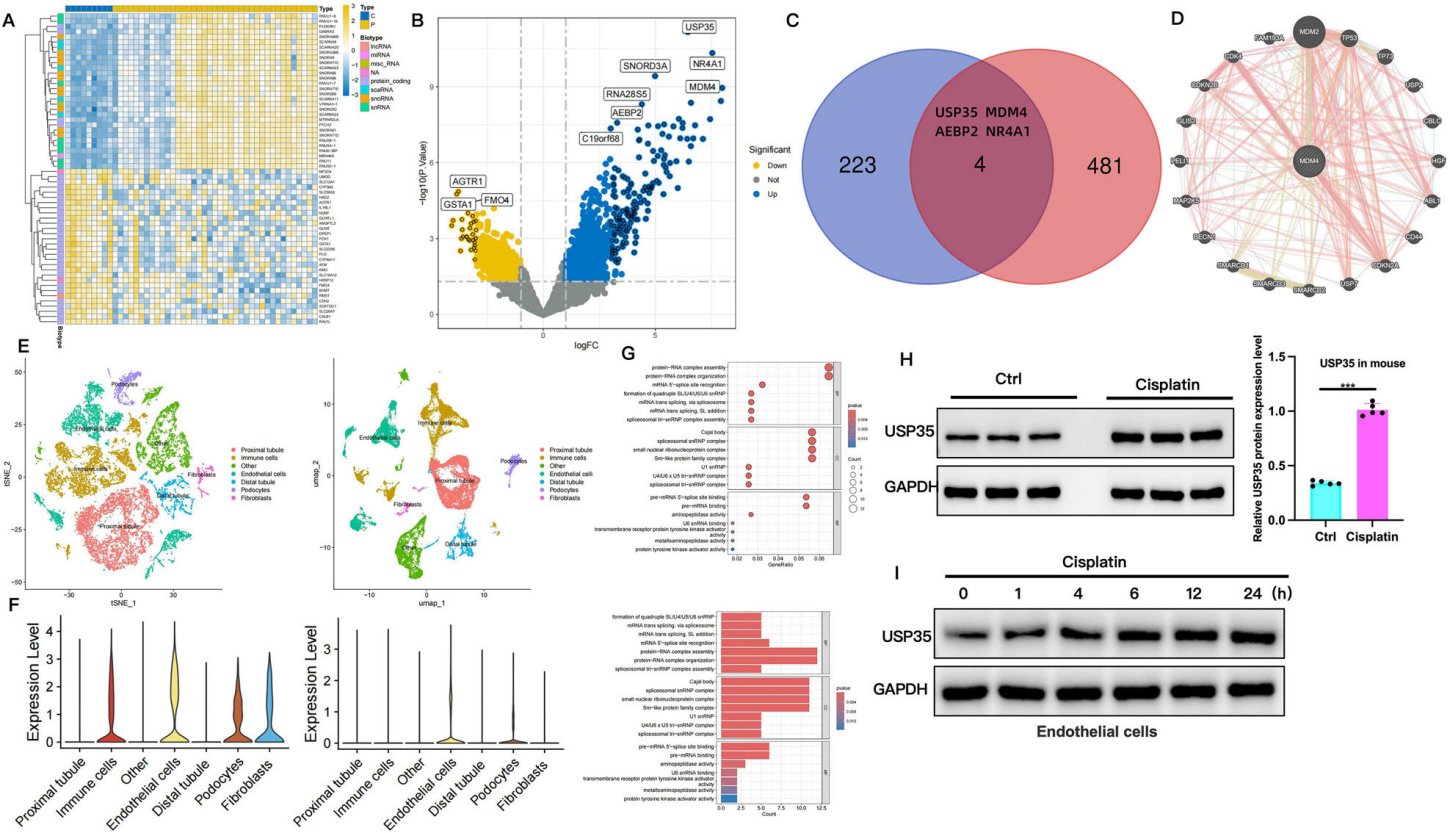
Upregulation of USP35 in AKI. **A** Heat map of differential gene analysis in AKI. USP35 is upregulated in AKI, accompanied by the expression of genes such as MDM4 and NR4A1. **B** The results of the differential gene volcano map analysis show that the upregulation of USP35 expression is the most significant, and it is also related to ferroptosis. **C** The Venn diagram results of AKI-ferroptosis analysis show that the four genes USP35, MDM4, AEBP2, and NR4A1 are associated with ferroptosis in AKI. **D** Analysis results of MDM4-related genes. Single-cell sequencing analysis results of (**E**, **F**): USP35 and MDM4 expression, USP35 and MDM4 are mainly enriched in endothelial cells. **G** GO and KEGG analysis. **H**, **I** cisplatin-induced upregulation of USP35 expression in AKI tissues and cisplatin-induced upregulation of USP35 expression in endothelial cells (*n* = 5). **H**, **I** cisplatin-induced upregulation of USP35 expression in AKI tissues and cisplatin-induced upregulation of USP35 expression in endothelial cells (*n* = 5). Each point represents an independent experiment. Group comparisons: ^ns^*P* > 0.05, ^*^P < 0.05, ^**^P < 0.01, ^***^P < 0.001.

We first analyzed the differentially expressed genes in acute kidney injury using the GEO database (GSE139061) and generated a heatmap [Fig. 1A]. Our analysis revealed that USP35 is upregulated in AKI, along with genes such as MDM4 and NR4A1 [Fig. 1B]. A Venn diagram analysis of ferroptosis-related and AKI-related genes showed that USP35, MDM4, AEBP2, and NR4A1 are associated with ferroptosis in AKI [Fig. 1C]. Network analysis of MDM4 revealed its close relationship with MDM2 and P53 [Fig. 1D]. Detection of USP35 showed that its expression level is significantly upregulated in cisplatin-induced AKI [Fig. 1H]. Analysis of single-cell data (GSE245446) indicated that USP35 and MDM4 are highly expressed and enriched in endothelial cells, with significant differences compared to immune cells [Fig. 1E, F]. GO and KEGG enrichment analysis showed that USP35 is associated with protein-protein interaction signaling [Fig. 1G]. Our results also showed that USP35 expression is significantly upregulated in endothelial cells following cisplatin induction [Fig. 1I].

### Kidney-specific USP35 knockout alleviates cisplatin-induced AKI and endothelial cell injury

**Fig. 2:**
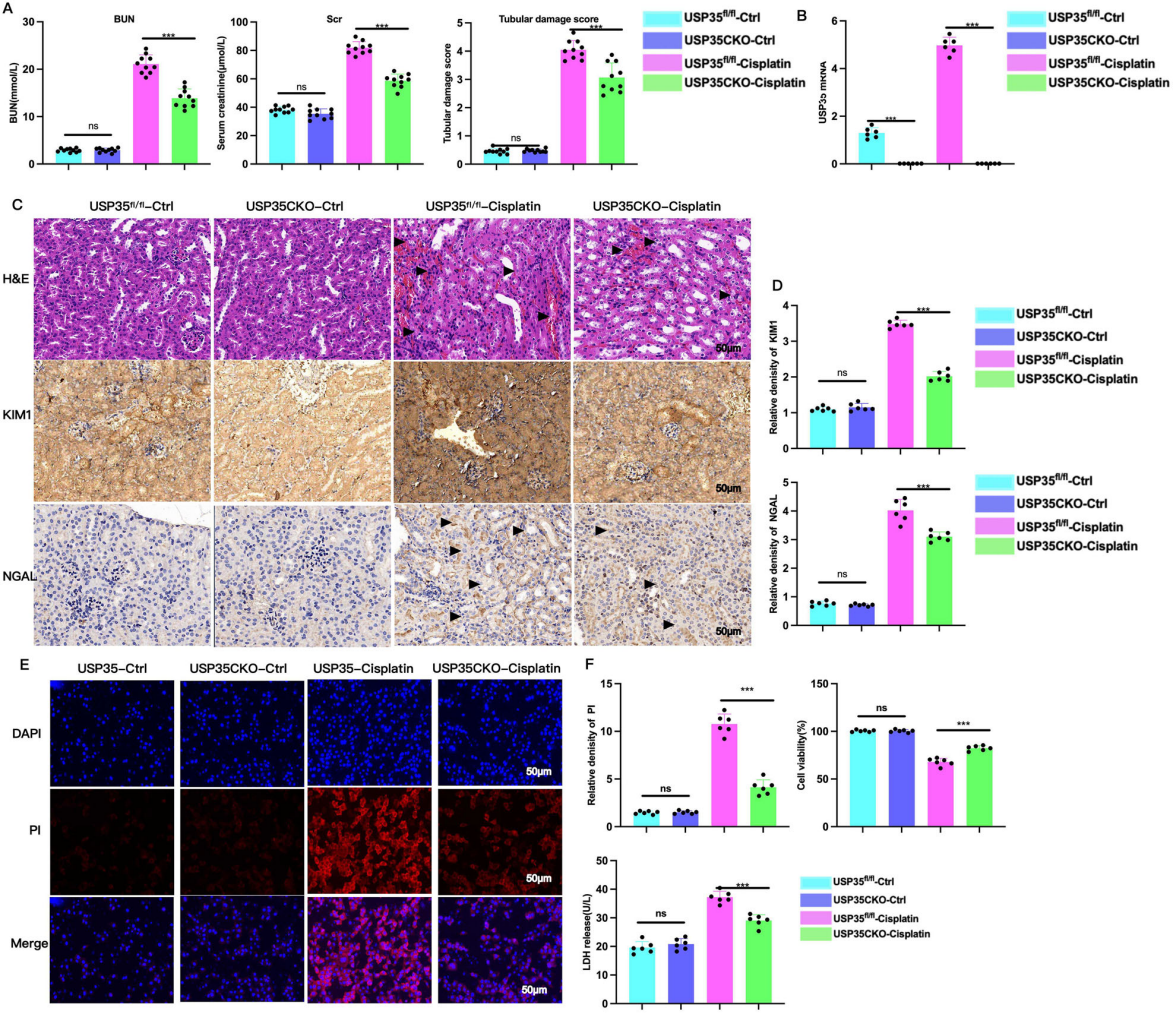
Kidney-specific USP35 knockout alleviates cisplatin-induced AKI and endothelial cell injury. **A** Renal function test indicators, BUN, Scr and renal tubular damage score results show that there is no significant difference between USP35^fl/fl^-Ctrl and USP35CKO-Ctrl, and the value of USP35fl/fl-Cisplatin is significantly higher than USP35CKO-Cisplatin (*n* = 10). **B** Determination results of USP35mRNA, (*n* = 6). **C**-**D** Histopathological evaluation, including H&E, KIM1, NGAL staining and quantitative analysis. KIM1 and NGAL are the main indicators of AKI, and the positive rate in USP35^fl/fl^-Cisplatin is significantly higher than USP35CKO-Cisplatin (*n* = 6). **E**, **F** Cell PI staining, cell viability, and LDH detection results. Compared with USP35-Ctrl, PI uptake in USP35^fl/fl^-Cisplatin is significantly increased, and the luminosity value is higher than Ctrl. At the same time, cell viability is down-regulated, and LDH release is increased. The PI luminosity value and LDH release degree in USP35CKO-Cisplatin are lower than USP35^fl/fl^-Cisplatin (*n* = 6). Each point represents an independent experiment. Group comparisons: ^ns^*P* > 0.05, ^*^P < 0.05, ^**^P < 0.01, ^***^P < 0.001.

In USP35^fl/fl^ and USP35CKO mice, our results showed that cisplatin induced renal injury, and the degree of renal functional impairment was significantly reduced in USP35CKO-Cisplatin compared to USP35^fl/fl^ -Cisplatin. Levels of BUN, Scr, and renal tubular injury scores showed no significant differences between USP35^fl/fl^ -Ctrl and USP35CKO-Ctrl, but USP35^fl/fl^ -Cisplatin had significantly higher values than USP35CKO-Cisplatin [Fig. 2A]. USP35 mRNA detection showed no expression in USP35CKO [Fig. 2B]. H&E staining revealed that cisplatin induced renal injury with obvious cell damage, inflammatory response, and edema, which was significantly alleviated in USP35CKO-Cisplatin compared to USP35^fl/fl^ -Cisplatin. KIM1 and NGAL, major indicators of AKI, had significantly higher positivity rates in USP35^fl/fl^ -Cisplatin [Fig. 2C, D].

In endothelial cell experiments, cisplatin induced cell damage. PI staining intensity and LDH release were low in USP35^fl/fl^ -Ctrl and USP35CKO-Ctrl, with no significant differences between groups. However, in USP35^fl/fl^ -Cisplatin, PI uptake was significantly increased, cell viability was decreased, and LDH release was elevated. In contrast, USP35CKO-Cisplatin had lower PI intensity and LDH release and higher cell viability compared to USP35^fl/fl^ -Cisplatin [Fig. 2E, F].

### Kidney-specific USP35 Knockout attenuates cisplatin-induced AKI and endothelial cell ferroptosis

**Fig. 3:**
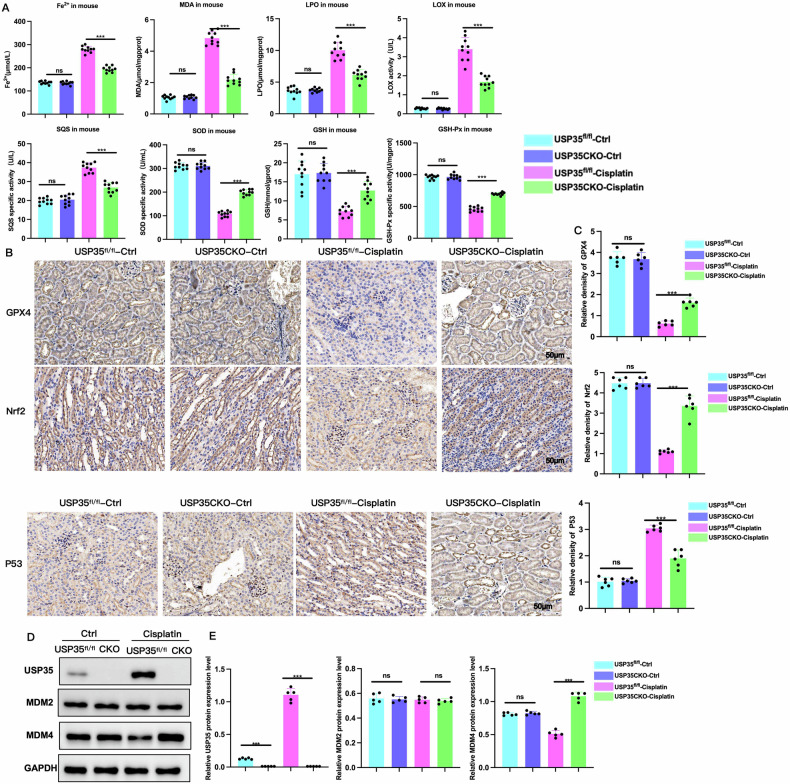
USP35 conditional knockout inhibits ferroptosis in cisplatin-induced AKI. **A** Test results of iron ions, MDA, lipid metabolism indicators (LPO, LOX, SQS) and oxidative damage indicators (SOD, GSH, GSH-Px) in tissues, USP35^fl/fl^-Ctrl and USP35CKO, There was no significant difference in the Ctrl comparison. The levels of ferrous iron ions, MDA, LPO, LOX, and SQS in USP35^fl/fl^-Cisplatin were significantly increased, while the levels of SOD, GSH, and GSH-Px were decreased (*n* = 10). **B**, **C** Histochemical staining and quantitative analysis results of GPX4, Nrf2, and P53. USP35^fl/fl^-Cisplatin in GPX4 and Nrf2 is significantly lower than USP35CKO-Cisplatin. Cisplatin can increase the expression of P53 (*n* = 6). **D**, **E** Relative protein expression level detection results, Cisplatin mainly affects the expression of MDM4, not MDM2, MDM2 has no significant change (*n* = 6). Each point represents an independent experiment. Group comparisons: ^ns^*P* > 0.05, ^*^P < 0.05, ^**^P < 0.01, ^***^P < 0.001.

In AKI tissues, there were no significant differences in ferrous ions, MDA, lipid peroxidation (LPO, LOX, SQS), and oxidative damage indicators (SOD, GSH, GSH-Px) between USP35^fl/fl^-Ctrl and USP35CKO-Ctrl. However, in USP35^fl/fl^-Cisplatin, levels of ferrous ions, MDA, LPO, LOX, and SQS were significantly increased, while SOD, GSH, and GSH-Px levels were decreased compared to Ctrl. In contrast, USP35CKO-Cisplatin had lower levels of ferrous ions, MDA, LPO, LOX, and higher levels of SOD, GSH, and GSH-Px compared to USP35^fl/fl^-Cisplatin [Fig. 3A]. We also detected the expression of ferroptosis-related proteins GPX4 and Nrf2. GPX4 and Nrf2 expression levels were high in Ctrl, and cisplatin reduced their expression. However, USP35^fl/fl^-Cisplatin had significantly lower levels than USP35CKO-Cisplatin, while P53 expression was higher in USP35^fl/fl^-Cisplatin [Fig. 3B, C]. Protein quantification showed that cisplatin mainly affected MDM4 expression, not MDM2. MDM2 levels remained unchanged, while MDM4 levels were significantly higher in USP35CKO [Fig. 3D, E].

In cisplatin-induced renal endothelial cell injury, ferroptosis was also activated. Detection of total and ferrous iron levels showed that levels were significantly higher in USP35-Cisplatin than in Ctrl, while USP35CKO-Cisplatin had lower levels than USP35-Cisplatin. Lipid peroxidation indicators LOX, SQS, and MDA were significantly higher in USP35-Cisplatin than in Ctrl, while USP35CKO-Cisplatin had lower levels. Oxidative damage indicators SOD, FSP-1, GSH, and GSH-Px were lower in USP35-Cisplatin than in Ctrl, while USP35CKO-Cisplatin had higher levels [Supporting Information Fig. S1A]. FerroOrange probe staining for iron ions showed low iron content in Ctrl, with no significant differences in fluorescence intensity. In contrast, USP35-Cisplatin had significantly higher iron content, while USP35CKO-Cisplatin had lower iron content than USP35-Cisplatin [Supporting Information Fig. S1B, C]. Protein detection also showed that USP35 affected MDM4 expression in renal epithelial cells, with no significant effect on MDM2 [Supporting Information Fig. S1D, E]. DCFH-DA probe staining for ROS showed low ROS expression and fluorescence intensity in Ctrl. In contrast, USP35-Cisplatin had significantly higher ROS expression and fluorescence intensity, while USP35CKO-Cisplatin had lower ROS expression [Supporting Information Fig. S1F, G]. Detection of ferroptosis-related proteins showed that GPX4, Nrf2, and HO-1 levels were significantly higher in USP35CKO-Cisplatin than in USP35-Cisplatin [Supporting Information Fig. S1H, I].

### Overexpression of USP35 promotes cisplatin-induced AKI and endothelial cell injury

We used AAV9-USP35 to overexpress USP35 in mouse renal tissues and endothelial cells, with EV as a control. After cisplatin treatment, renal injury and endothelial cell damage were significantly higher in Cisplatin+USP35^oe^ than in Cisplatin+EV. Levels of BUN, Scr, and renal tubular injury scores were significantly higher in Cisplatin+USP35^oe^ than in Cisplatin+EV, and USP35 mRNA levels were also higher in Cisplatin+USP35^oe^ [Supporting Information Fig. S2A]. Histopathological staining of renal tissues showed that KIM1 and NGAL levels were significantly higher in Cisplatin+USP35^oe^, and the tissues exhibited obvious necrosis and damage [Supporting Information Fig. S2B, C]. PI staining of endothelial cells showed significantly higher PI fluorescence intensity in Cisplatin+USP35^oe^ than in Cisplatin+EV [Supporting Information Fig. S2D, E]. Cell viability and LDH detection showed that Cisplatin+USP35^oe^ had significantly lower cell viability and higher LDH release than Cisplatin+EV [Supporting Information Fig. S2F, G].

### Overexpression of USP35 promotes cisplatin-induced AKI and endothelial cell ferroptosis

**Fig. 4:**
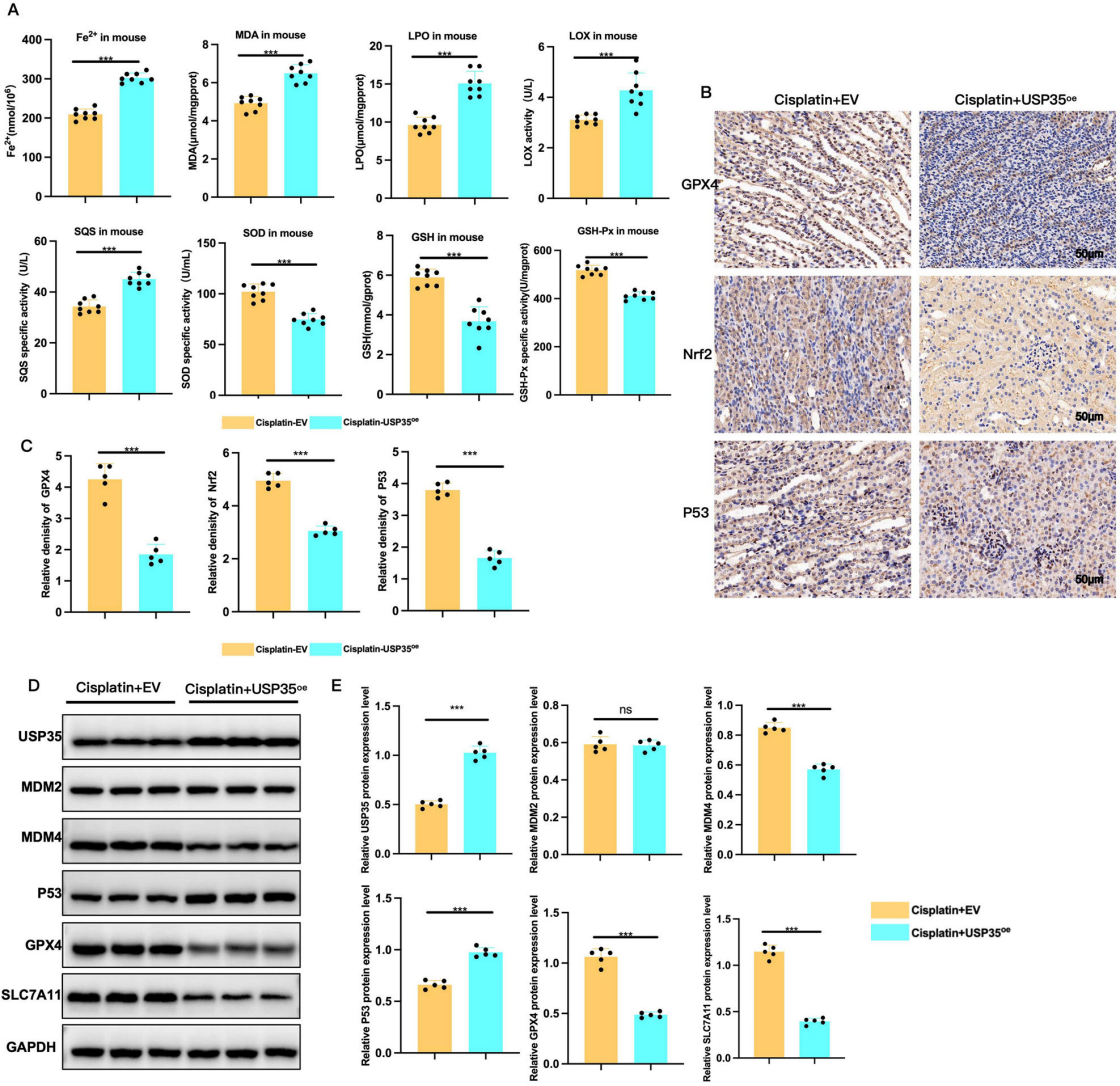
Overexpression of USP35 promotes ferroptosis in cisplatin-induced AKI. **A** Test results of iron ions, MDA, lipid metabolism indicators (LPO, LOX, SQS) and oxidative damage indicators (SOD, GSH, GSH-Px) in tissues. The levels of ferrous iron ions, MDA, and lipid metabolism LPO, LOX, and SQS in AKI are significantly higher than USP35 + EV, while the antioxidant factors SOD, GSH, and GSH-Px are lower than Cisplatin+EV (*n* = 10). **B**-**C** Histochemical staining and quantitative analysis results of GPX4, Nrf2, and P53. The levels of Cisplatin+USP35^oe^ are lower than Cisplatin+EV (*n* = 6). **D**, **E** Relative protein expression level detection results. Overexpression of USP35 does not affect the level of MDM2, but the levels of MDM4 and GPX4 are significantly reduced, while the levels of P53 is significantly increased (*n* = 6). Each point represents an independent experiment. Group comparisons: ^ns^ *P* > 0.05, ^*^P < 0.05, ^**^P < 0.01, ^***^P < 0.001.

After overexpressing USP35 and treating with cisplatin, we detected ferroptosis-related indicators in tissues and cells. In AKI, levels of ferrous ions, MDA, and lipid peroxidation (LPO, LOX, SQS) were significantly higher in Cisplatin+USP35^oe^ than in Cisplatin+EV, while antioxidant factors SOD, GSH, and GSH-Px were lower in Cisplatin+USP35^oe^ [Fig. 4A]. Histochemical staining of GPX4, Nrf2, and P53 showed that levels were lower in Cisplatin+USP35^oe^ than in Cisplatin+EV [Fig. 4B, C]. Protein detection showed that USP35 overexpression did not affect MDM2 levels but significantly decreased MDM4 and GPX4 levels while increasing P53 levels [Fig. 4D, E].

**Fig. 5:**
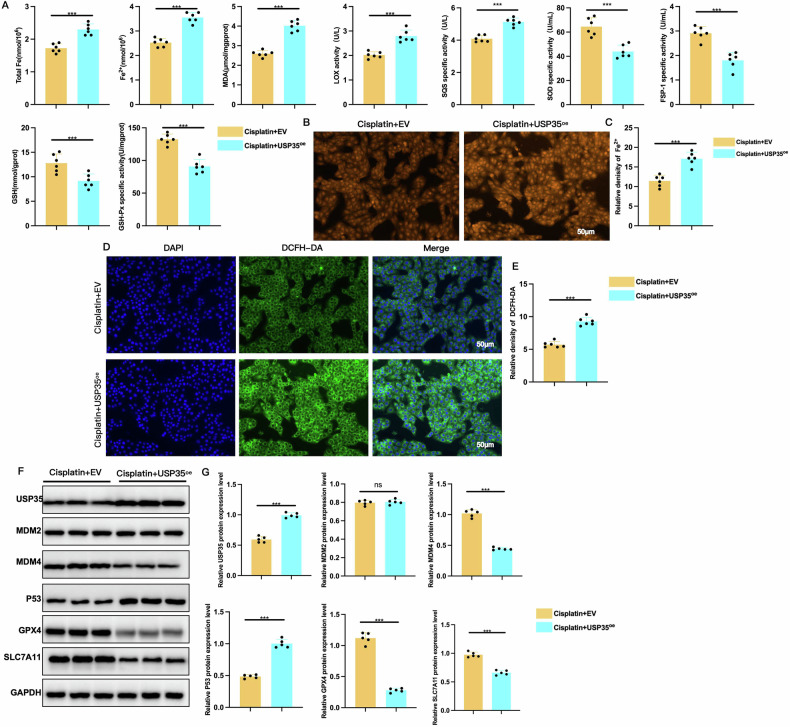
Overexpression of USP35 promotes ferroptosis in cisplatin-induced endothelial cells. **A** Test results of iron ions, MDA, lipid metabolism indicators (LPO, LOX, SQS) and oxidative damage indicators (SOD, FSP-1, GSH, GSH-Px) in tissues, Cisplatin+USP35oe2. The levels of valence iron ions, MDA, and lipid metabolism LPO, LOX, and SQS were significantly higher than Cisplatin+EV, while the antioxidant factors SOD, GSH, and GSH-Px were lower than Cisplatin+EV (*n* = 6). **B**, **C** Staining results and fluorescence intensity analysis of FerroOrange probe to detect iron ions. The iron ion content of Cisplatin+USP35oe is significantly higher than that of Cisplatin+EV (*n* = 6). **D**, **E** Staining results and fluorescence intensity analysis of ROS detected by DCFH-DA probe. The ROS level of Cisplatin+USP35oe is significantly higher than that of Cisplatin+EV (*n* = 6). **F**, **G** Protein expression level detection results of ferroptosis-related proteins GPX4, Nrf2, and HO-1, (*n* = 6). Each point represents an independent experiment. Group comparisons: ^ns^*P* > 0.05, ^*^P < 0.05, ^**^P < 0.01, ^***^P < 0.001.

Overexpression of USP35 in endothelial cells also promoted cell ferroptosis. Detection of ferroptosis indicators in cells showed that levels of ferrous ions, MDA, and lipid peroxidation (LPO, LOX, SQS) were significantly higher in Cisplatin+USP35^oe^ than in Cisplatin+EV, while antioxidant factors SOD, FSP-1, GSH, and GSH-Px were lower in Cisplatin+USP35^oe^[Fig. 5A]. FerroOrange probe staining for iron ions showed significantly higher iron content in Cisplatin+USP35^oe^ than in Cisplatin+EV [Fig. 5B, C]. DCFH-DA probe staining for ROS showed significantly higher ROS levels in Cisplatin+USP35^oe^ than in Cisplatin+EV [Fig. 5D, E]. Protein detection showed that USP35 overexpression did not affect MDM2 levels but significantly decreased MDM4 and GPX4 levels while increasing P53 levels [Fig. 5F, G].

### USP35 binds to MDM4 and promotes its autophagic degradation

**Fig. 6:**
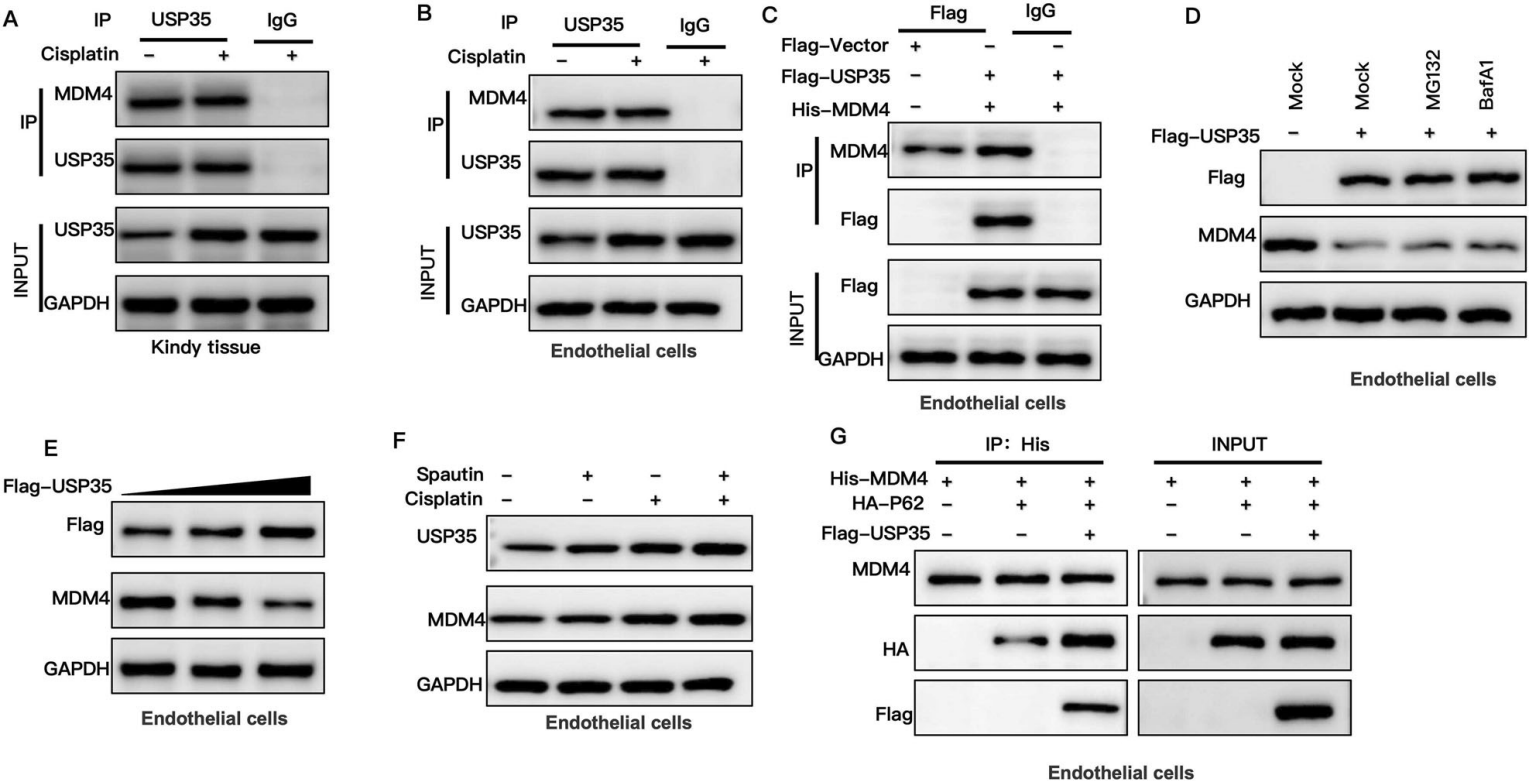
USP35 binds to MDM4 and promotes its autophagic activation and degradation. **A** Co-IP analysis of USP35 and MDM4 in endothelial cells treated with cisplatin. **B** Co-IP analysis of USP35 and MDM4 in AKI tissues treated with cisplatin. **C** Co-IP of USP35 and MDM4 in endothelial cells co-transfected with Flag-USP35 and His-MDM4. **D** Representative immunoblot images of Flag-USP35 and MDM4 in endothelial cells transfected with Flag-EV or Flag-USP35 and treated with Mock, MG132, and Bafilomycin A1. **E** Immunoblot of USP35 and MDM4 in endothelial cells transfected with Flag-USP35. **F** Representative immunoblot images of USP35 and MDM4 in endothelial cells treated with Spautin-1 and then cisplatin. **G** Co-IP of MDM4, P62, and Flag-USP35 in endothelial cells co-transfected with His-MDM4, HA-P62, and Flag-USP35.

We first verified the endogenous interaction of USP35 and MDM4 proteins in renal tissues and endothelial cells, and the results demonstrated the interaction of USP35-MDM4 in tissues and cells, [Fig. 6A, B]. The Co-IP results further confirmed the exogenous interaction between USP35 and MDM4 in endothelial cells co-transfected with flag-USP35 and HIS-MDM4 [Fig. 6C], and these results demonstrated the existence of interaction between USP35 and MDM4. We studied the influence of USP35 on the stability of MDM4. Overexpression of USP35 in endothelial cells leads to a decrease in MDM4 expression. The use of proteasome inhibitor MG132 and autophagy inhibitor BafA1 can increase the protein level of MDM4, indicating that USP35 exerts MDM4 degradation through autophagy [Fig. 6E]. The alteration of MDM4 is regulated post-transcriptional, and subsequently, We used the specific inhibitor Spautin-1 and showed similar results. The inhibitory effect of USP35 could increase the level of MDM4 [Fig. 6F]. To explore the degradation of MDM4 protein mediated by USP35, endothelial cells expressing flag-USP35 were treated with proteasome inhibitors or autophagy inhibitors. Proteasome inhibitors (MG132) had no effect on USP35-mediated degradation of MDM4, while autophagy inhibitors (BafA1) specifically inhibited the degradation of MDM4, indicating the involvement of the autophagy-lysosomal pathway, [Fig. 6G]. The interaction between the key autophagy mediator P62 and proteins can promote protein degradation by activating autophagy. We speculate that USP35 may promote autophagolysosome-related degradation of MDM4 in a P62-dependent manner. We examined the effect of USP35 on the binding of P62 and MDM4. The results showed that overexpression of USP35 promoted the binding of MDM4 and P62 in endothelial cells, [Fig. 6D]. The interaction between MDM4 and P62 was also detected in cultured endothelial cells and kidney tissues [Supporting Information Fig. S3A, B].

### USP35 Regulates MDM4 K63 Deubiquitination

**Fig. 7:**
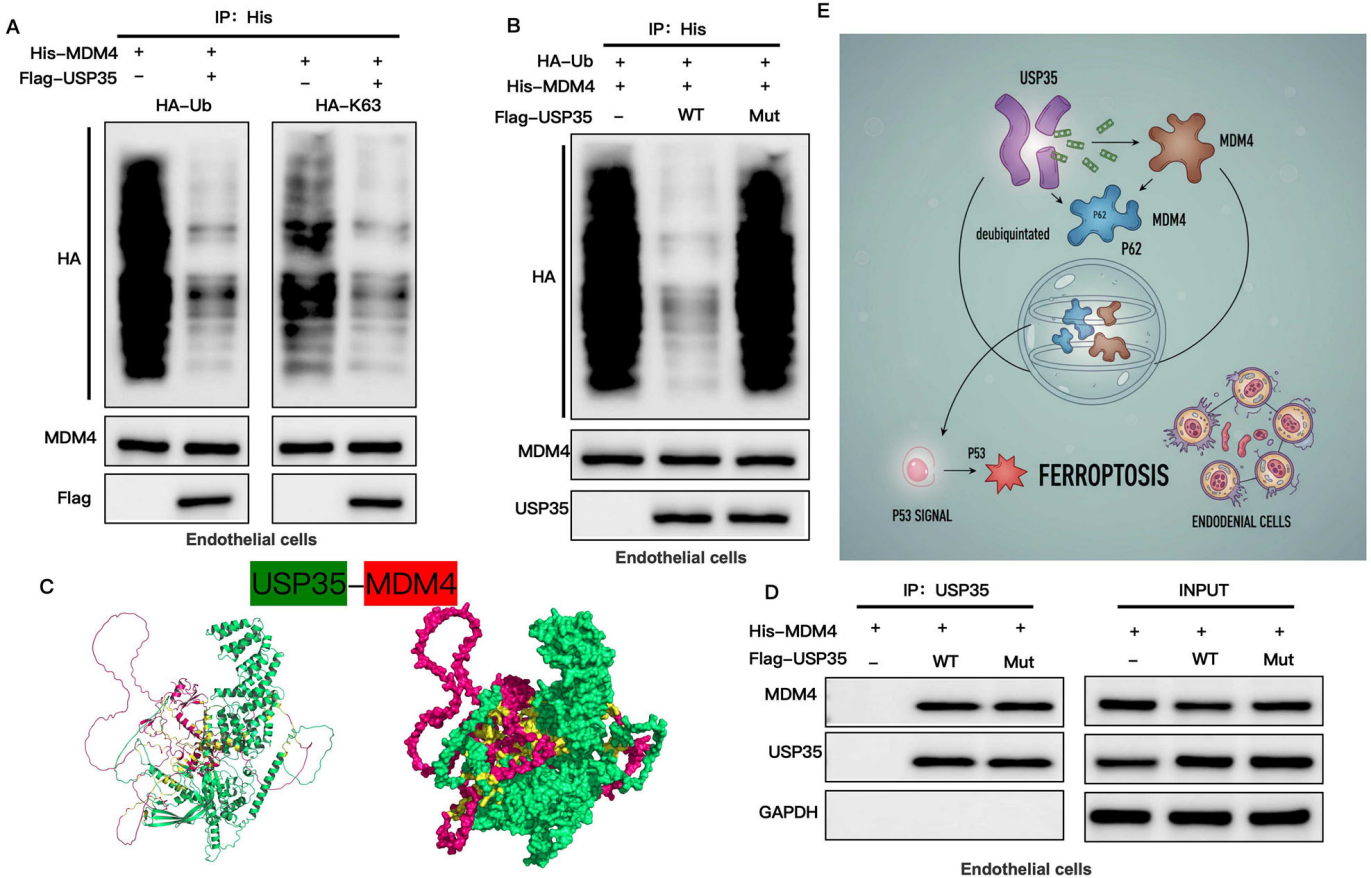
USP35 regulates K63 deubiquitination of MDM4. **A** Immunoprecipitation of His-MDM4 with anti-His antibody in endothelial cells co-transfected with His-MDM4, HA-Ub, or HA-K63 and treated with Flag-USP35 and Baf A1. **B** Immunoprecipitation of His-MDM4 with anti-His antibody in endothelial cells co-transfected with His-MDM4 and HA-Ub and treated with Flag-USP35-WT or Flag-USP35-Mut and Baf A1. **C** Interaction and binding sites of USP35 and MDM4. **D** Immunoprecipitation of His-MDM4 with anti-His antibody in endothelial cells co-transfected with His-MDM4 and HA-Ub and treated with Flag-USP35-WT or Flag-USP35-Mut and Baf A1. **E** Schematic diagram of USP35 regulating K63-linked deubiquitination of MDM4 and promoting its degradation via P62-mediated autophagy.

Next, we investigated the effect of USP35 on MDM4 deubiquitination. Co-transfection of Flag-USP35, HA-Ub/K63, and His-MDM4 in endothelial cells showed that USP35 removed ubiquitin chains from MDM4, especially K63-linked ubiquitin chains [Fig. 7A]. USP35 regulated K63-linked deubiquitination of MDM4 in endothelial cells [Supporting Information Fig. S4A]. K63-linked ubiquitin chains mainly affect protein-protein interactions, suggesting that USP35-mediated K63-linked deubiquitination of MDM4 might promote the binding of P62 and MDM4 [Supporting Information Fig. S4B]. We performed protein interaction studies between MDM4 and USP35 [Fig. 7B]. We also constructed a mutant type Mut. The USP35-Mut mutant could still bind to MDM4, similar to its wild-type homolog (USP35-WT). However, compared to USP35-WT, the USP35-Mut mutant could not remove ubiquitin molecules from MDM2 [Fig. 7C]. In summary, USP35 regulates K63-linked deubiquitination of MDM4 through its active site, thereby promoting MDM4 degradation via P62-mediated autophagy [Fig. 7D].

## Discussion

Ferroptosis is a newly discovered non-apoptotic form of regulated cell death, characterized by iron-dependent lipid peroxidation and ROS accumulation [18]. P53 is a tumor suppressor gene, and recent studies have shown that P53 can also regulate ferroptosis by affecting iron metabolism [19–21], polyunsaturated fatty acid metabolism [22, 23], amino acid metabolism, and NADPH-mediated cellular metabolic processes [24, 25], playing a role in the pathogenesis of kidney diseases [26, 27]. In AKI, P53 is activated and inhibits the expression of SLC7A11, inducing ferroptosis in damaged tissues and worsening renal injury [28]. The P53 ferroptosis pathway can serve as a target for the prevention and treatment of AKI [29]. P53 can also inhibit SLC7A11, inducing ferroptosis in damaged tissues after AKI. After AKI, the accumulation of Fe^2+^ triggers the Fenton reaction, leading to increased ROS and ultimately lipid peroxidation and cell ferroptosis [30]. In a folic acid-induced AKI model, upregulation of P53 expression inhibited the expression of SLC7A11, reducing GSH biosynthesis, inducing renal cell ferroptosis, and disrupting cellular redox homeostasis, resulting in extensive renal tubular necrosis and inflammation [31]. Therefore, based on existing reports, P53-mediated ferroptosis plays an important role in AKI. Activation of P53 is regulated by multiple signaling pathways, such as the MDMs family, which is directly related to P53 activation. It has been reported that MDM2 can inhibit P53 activation at the ubiquitination level [32], while MDM4 inhibits P53 activation at the transcriptional level [33]. The reason is that the gene encoding MDM4 contains a P53-binding domain at the N-terminus and a ring finger domain at the C-terminus, similar to the structure of P53-binding proteins [34, 35].In our study, we found that in cisplatin-induced AKI, the expression of MDM4 was downregulated, while MDM2 remained unchanged, indicating that MDM4 plays a major role in AKI. Downregulation of MDM4 leads to P53 activation and subsequent induction of ferroptosis.

Cisplatin is a common chemotherapeutic drug with significant nephrotoxicity, easily causing renal injury in clinical settings, accompanied by damage to renal tubules and epithelial cells. Through analysis, we found that USP35 is highly expressed in cisplatin-induced AKI [36, 37]. USP35 is a deubiquitinase that can modify proteins post-transcriptionally. Existing studies have shown that multiple deubiquitinases have clear roles in renal injury. For example, JOSD2 can effectively prevent renal tubular injury and inflammation in AKI mice. SIRT7 is a potential substrate of JOSD2. JOSD2 removes K63-linked ubiquitination from SIRT7 through its active site C24 and promotes p62-mediated autophagic degradation of SIRT7, thereby inhibiting the phosphorylation and nuclear translocation of p65 and reducing the inflammatory response of renal tubular epithelial cells [15]. OTUD1 binds to CDK9 and induces deubiquitination of CDK9, thereby inducing inflammatory responses and fibrosis in renal epithelial cells [16]. Our results showed that USP35 can interact with MDM4 through deubiquitination. After MDM4 is deubiquitinated, it further binds to p62, activating the autophagy signal and promoting the autophagic degradation of MDM4.

Epithelial cell injury is one of the main pathological processes in AKI. Our analysis showed that USP35 and MDM4 are mainly enriched in epithelial cells. Experimental results showed that USP35 expression was upregulated in cisplatin-induced epithelial injury, while P53 was activated, and MDM4 expression was downregulated. Cisplatin induced ferroptosis in endothelial cells, as confirmed by lipid metabolism and oxidative damage indicators. Probe detection of iron ions and ROS levels also showed significant increases in iron ions and ROS content. In our mechanistic studies, we found the interaction between USP35 and MDM4, as well as the promotion of MDM4 K63-linked deubiquitination by USP35 through p62. K63-linked deubiquitination is one of the main ubiquitination forms, affecting the activation of substrate proteins. DUBs can inhibit substrate protein degradation by removing ubiquitin molecules from target proteins. Some DUBs can also promote substrate protein degradation through autophagy-dependent or other non-canonical pathways [36, 37]. Our study revealed that USP35 promotes lysosome-related degradation of MDM4 to inhibit the activation of the P53 signaling pathway. In animal models, we used USP35CKO and AAV9-USP35 to knock out or overexpress USP35, respectively. The results showed that USP35CKO can inhibit P53-induced ferroptosis and renal injury in AKI, while USP35 overexpression promotes ferroptosis and AKI renal injury. The in vivo and in vitro experimental results were consistent, proving the role of the USP35-MDM4-P53 signaling pathway in AKI.

Although we have identified the role of USP35-MDM4-P53 in AKI, there are still some limitations. For example, USP35 has been reported to bind to other potential substrate proteins. Although USP35 is enriched in endothelial cells, it is also expressed in other cells, including immune cells and renal tubular cells, and its role in these cells needs further investigation. Additionally, the role of MDM4-P53 in ferroptosis needs to be explored more deeply. Future studies should provide a more comprehensive understanding of the role of USP35 and other DUBs in AKI.

## Conclusion

In cisplatin-induced AKI, USP35 and MDM4 are enriched in epithelial cells. USP35 promotes P62-mediated autophagic degradation of MDM4 through deubiquitination. Degradation of MDM4 can promote P53-mediated ferroptosis, thereby worsening AKI progression. The USP35-MDM4-P53 pathway is a novel signaling pathway in cisplatin-induced AKI, and USP35 has the potential to be a new therapeutic target for AKI.

## Materials and Methods

### Main reagents

Cisplatin (purity ≥ 99%, MCE), HE staining solution (NO: YT8681, Beijing Yitai Biotechnology Co., Ltd.), Mouse serum urea nitrogen BUN and creatinine Scr detection kits (C013-2-1, C011-2-1, Nanjing Jiancheng Bioengineering Institute), PI + DAPI staining kit (ID22502, Solarbio), LDH and CCK-8 detection kits (BC0685, CA1210, Solarbio), Total iron ion detection kit, Ferrous ion detection kit, MDA, LOX, SQS, SOD, FSP-1, GSH, GSH-Px detection kits (Elabscience), FerroOrange probe and DCFH-DA probe (HYD0941, HYD0819, MCE), Targeted KIM1, NGAL monoclonal antibodies (histochemical staining 1:200, Elabscience), Targeted USP35, MDM2, MDM4, P53, GPX4, SLC7A11 monoclonal antibodies (Western-Blotting 1:1000, Proteintech), Plasmids encoding USP35 (mouse), USP35--Mut-E63 (mouse), MDM4 (mouse), p62 (mouse), HA--Ub (mouse), HA--K63 (mouse), and HA--K48 (mouse) were provided by GeneChem.

### Animal models

Animal care and experimental methods comply with the “Guide for the Care and Use of Laboratory Animals” published by the National Institutes of Health and were approved by the Jiaxing University Animal Care and Use Committee. Male C57BL/6 J mice (8 weeks old, 23–25 g) were purchased from Nanjing Model Animal Research Institute (Nanjing, China) and acclimated for 1 week before the experiment. All mice were housed in specific pathogen-free, environmentally controlled (temperature: 20–25 °C; humidity: 50 ± 5%) barrier conditions in individually ventilated cages.

An AKI model in wild-type C57BL/6 mice was established using cisplatin. Cisplatin (20 mg/kg/day) was administered intraperitoneally for 3 days to build the mouse AKI model [38, 39]. Blood samples and kidney tissues were collected from the mice for analysis after the end of the treatment.

A kidney-specific USP35 knockout mouse model (USP35CKO) was created by crossing USP35^fl/fl^ mice with mice expressing kidney-specific Cre recombinase. This model was constructed by Nanjing Model Animal Research Institute (Nanjing, China). The construction of the AKI model was consistent with the above.

Renal epithelial cell-specific overexpression of USP35 (USP35^oe^) was achieved through recombinant adeno-associated virus serotype 9 (AAV9). AAV9s carrying the kidney-specific promoter SGLT2 (SGLT2--3Flag--T2A--EGFP) and USP35 cDNA were prepared by GeneChem. In USP35^fl/fl^ mice, 100 μL of physiological saline containing 2 × 10^11^v.g. AAV9 virus was injected through the tail vein.

### Renal function detection [40]

The levels of BUN and Scr in mouse blood were detected. After extracting serum from fresh mouse blood, the creatinine detection kit and urea nitrogen detection kit instructions were followed to measure absorbance at 580 nm and 515 nm, respectively. The values of BUN and Scr were obtained based on the standard curve.

### Histopathological detection

H&E staining and histochemical staining of KIM1 and NGAL in mouse kidney tissues were performed to assess renal histopathological changes. H&E staining: The tissues were fixed in 10% formalin, dehydrated, and embedded, then sectioned continuously at a thickness of 5 μm. According to the manufacturer’s instructions (Solarbio, China), kidney sections were stained with hematoxylin and eosin (H&E) to examine the histological morphology of the kidney tissue.

### Histochemical staining

Immunohistochemistry was used to detect the expression of renal injury markers KIM1 and NGAL, as well as GPX4, Nrf2, and P53. Kidneys were fixed in 4% paraformaldehyde, dehydrated, embedded in paraffin, and sectioned. After dewaxing, antigen retrieval, and treatment with 3% hydrogen peroxide, the sections were blocked with BSA. Rabbit anti-KIM1, NGAL monoclonal antibodies (Bioword, China), rabbit anti-STAT1, CD86 monoclonal antibodies (Abcam, USA) were used, and the antibodies were diluted at KIM1 (1:200), NGAL (1:200), GPX4 (1:150), Nrf2 (1:150), P53 (1:200) and incubated overnight at 4 °C. The sections were washed with PBS, then horseradish peroxidase-conjugated secondary antibody was added to the sections and incubated at room temperature for 1 h. After washing 3 times with PBS for 5 min each, the sections were visualized with DAB, counterstained with hematoxylin, and mounted for microscopy.

### Cell Models

Mice Renal epithelial cells were isolated and identified by Purocell Biotechnology. The epithelial cells were cultured in DMEM with 10% FBS and 1% streptomycin and penicillin. The cells were cultured in a humidified incubator at 37 °C with 5% CO_2_. Renal epithelial cell injury was induced by treating the cells with 10 μmol/L cisplatin for 24 h to establish the injury model.

### Cell Viability

The viability of renal epithelial cells was assessed using the CCK-8 assay. 5000 cells per well were seeded in 96-well plates and treated as described. After discarding the culture medium, 100 μL of fresh medium containing 90 μL of fresh medium and 10 μL of CCK-8 solution was added to each well and incubated for 1 h. Absorbance was measured at 450 nm using a microplate reader, with a reference wavelength of 650 nm.

### LDH

The levels of LDH in the cell culture supernatant were measured according to the LDH detection kit instructions.

### Cell fluorescence staining

Renal epithelial cells were stained with PI-DAPI, FerroOrange probe for ferrous ions, and DCFH-DA probe. The cells were stained according to the kit instructions and observed under an inverted fluorescence microscope.

### Ferroptosis indicator detection

Ferroptosis indicators in tissues and cells, including total iron ions, ferrous ions, MDA, LOX, SQS, SOD, FSP-1, GSH, GSH-Px, were measured using the respective kits according to the instructions and quantified using standard curves.

### Western-blotting and Co-IP

Kidney tissues and cells were lysed using RIPA lysis buffer, and protein concentration was determined by BCA assay. Proteins were separated by SDS-PAGE and transferred to PVDF membranes. After blocking the membranes with 5% skim milk for 1.5 h, the membranes were incubated with primary antibodies overnight. The membranes were then treated with HRP-conjugated secondary antibodies for 1 h. The ECL chemiluminescent detection reagent was applied to the membranes, and the signals were visualized using a gel imaging system. For co-immunoprecipitation experiments, cell or tissue lysates were incubated with the specified antibodies (1:200) at 4 °C overnight, with total lysates serving as input controls. Subsequently, the lysates were immunoprecipitated with Protein A + G Agarose beads at 4 °C for 2–4 h. After washing three times with PBS, the immunoprecipitated samples were analyzed by Western blot.

### Real-time fluorescent quantitative PCR

Total RNA was extracted using TRIzol reagent. The isolated RNA was reverse-transcribed into cDNA using the HiScript III All-in-one RT SuperMix Perfect for qPCR kit. The cDNA was then subjected to RT-qPCR using the ChamQ Universal SYBR qPCR Master Mix. The primers used in this study (Supporting Information Table 1) were purchased from GenePharma.

### Statistical methods

Continuous data in this study are presented as mean ± standard error of the mean (SEM). Student’s t-test was used to compare differences between two groups, while one-way ANOVA with Bonferroni correction was used for comparisons among more than two groups. When significant differences were identified by ANOVA, Bonferroni correction was applied for post hoc multiple comparisons to determine specific group differences. Statistical analyses were performed using GraphPad Pro Prism 8.0 (GraphPad, San Diego, USA). All analyses in this study were considered statistically significant at *P* < 0.05.

## Supplementary information

WB

Supporting Information

## Data Availability

The data that support the findings of this study are available from the corresponding author upon reasonable request.
