# Supplementary figures and images for: Deubiquitinase USP35 regulates MDM4 degradation to promote endothelial ferroptosis and renal injury progression
Source: Cell Death Discov. 2026 May 25;12:314. doi: 10.1038/s41420-026-03128-5 (PMC13385968; doi:10.1038/s41420-026-03128-5)

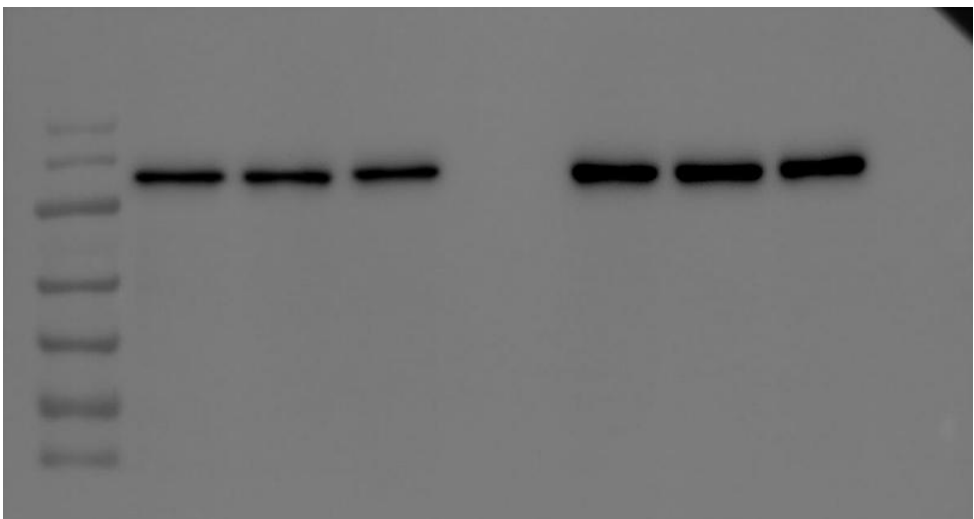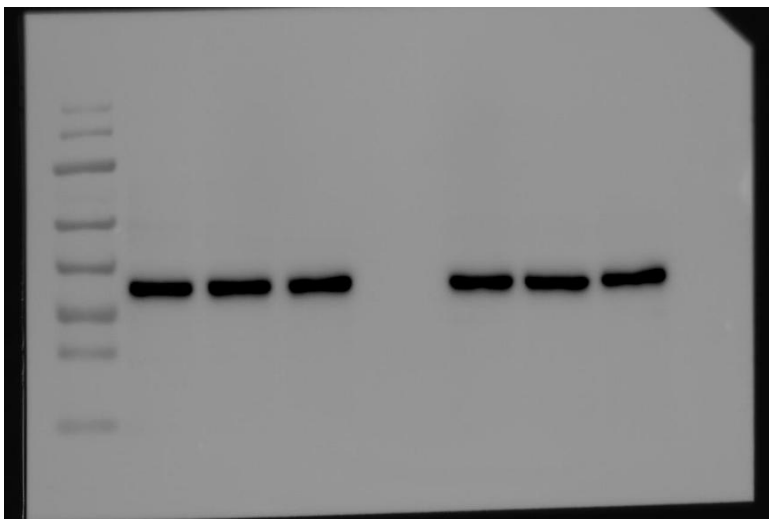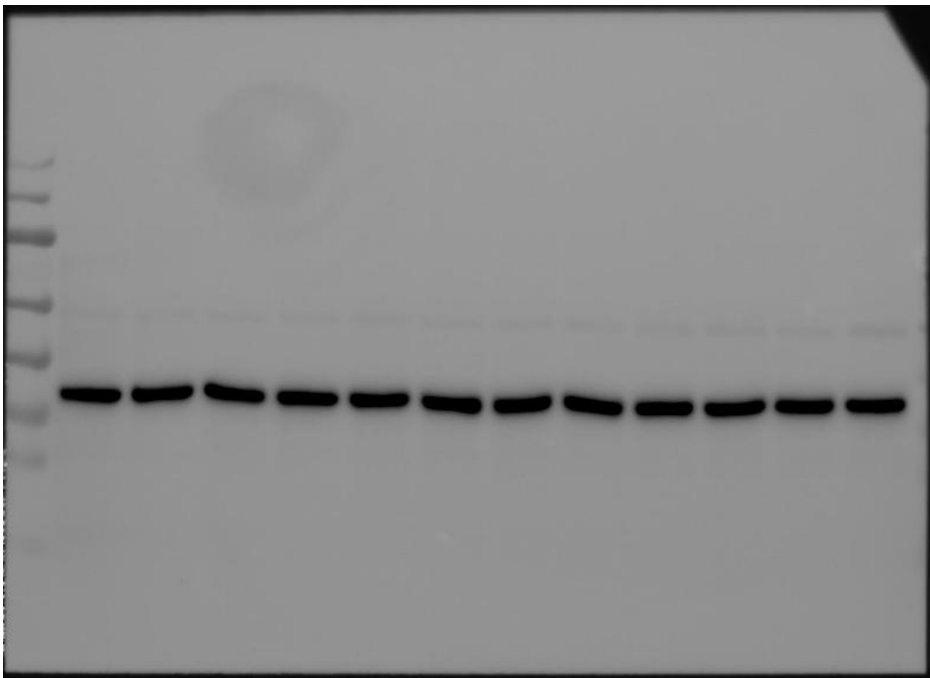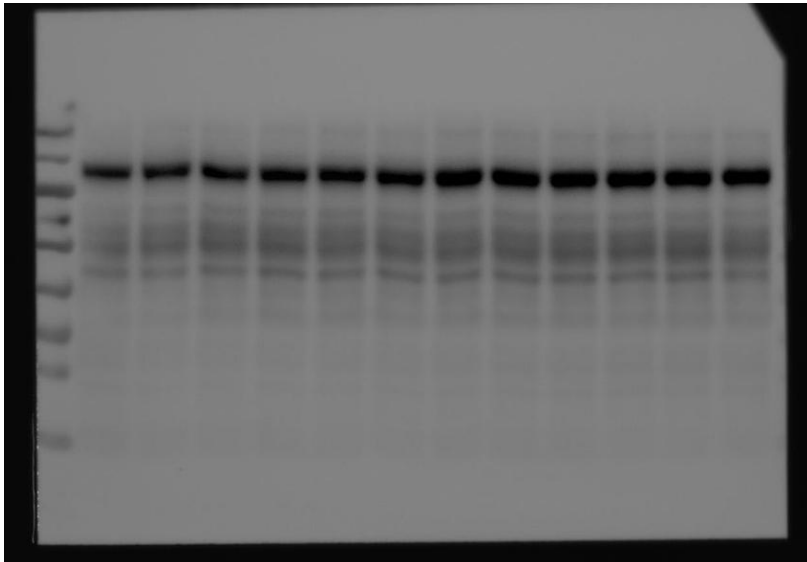

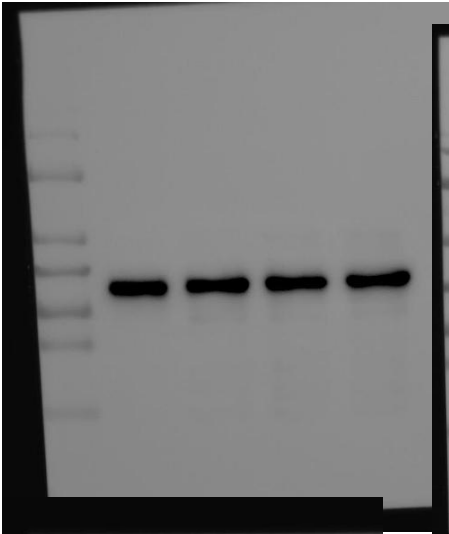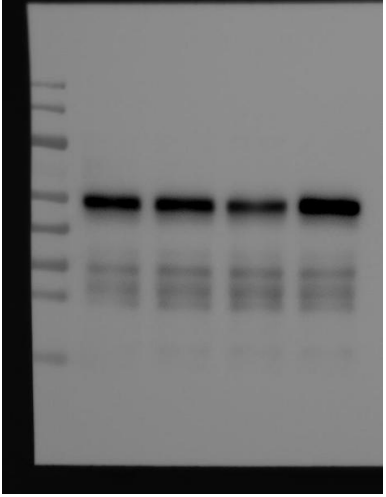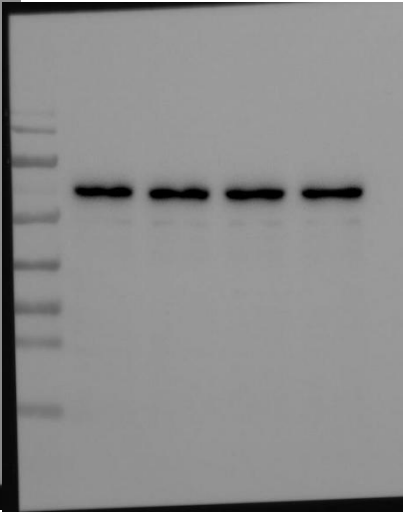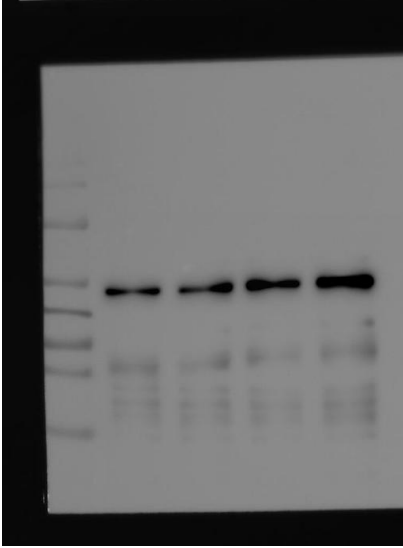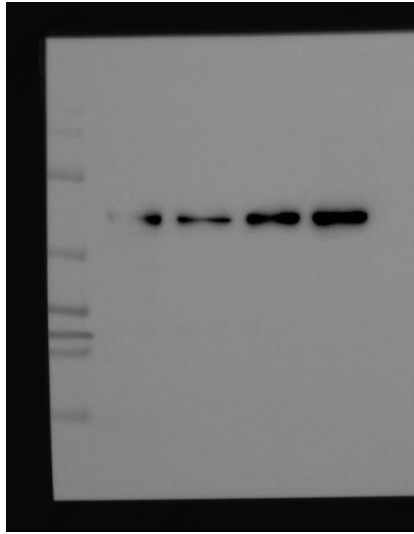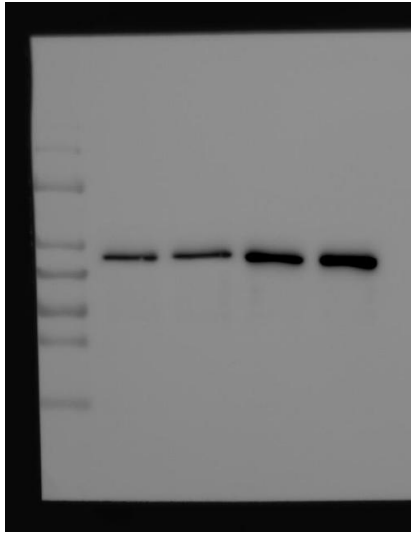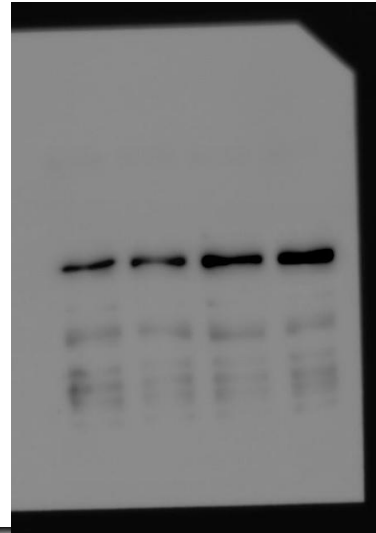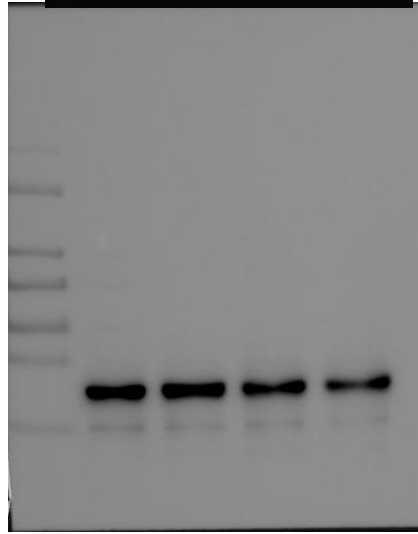

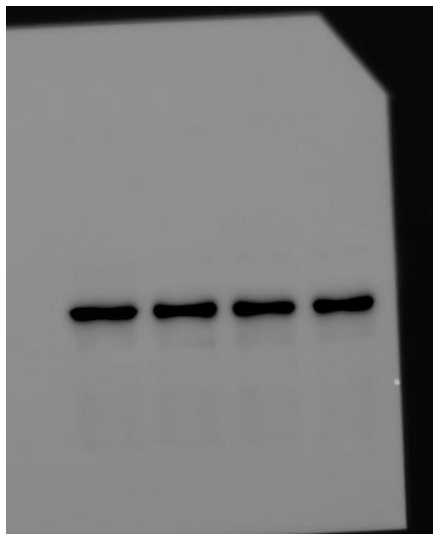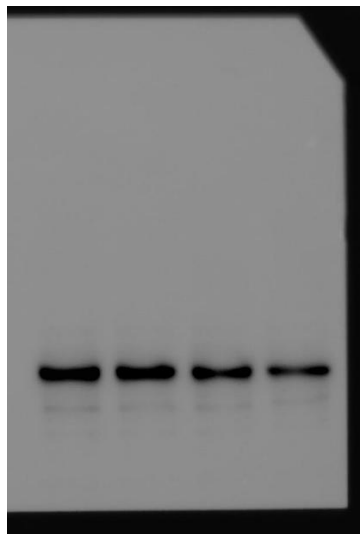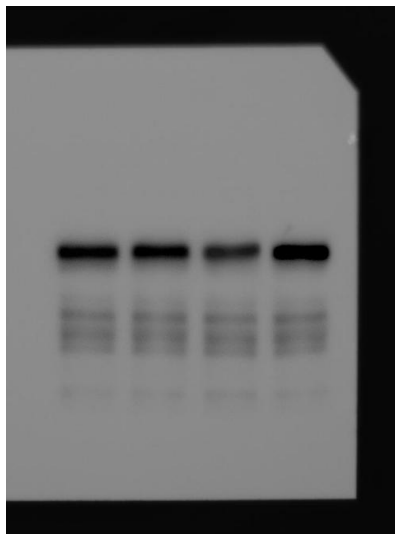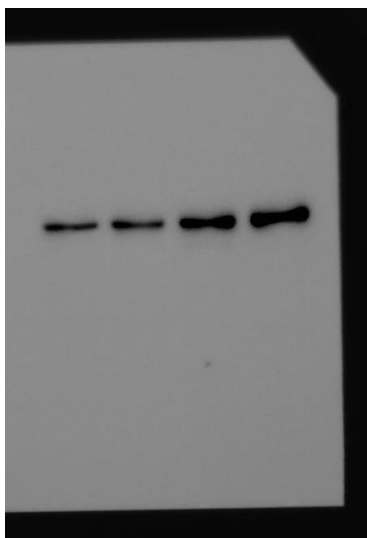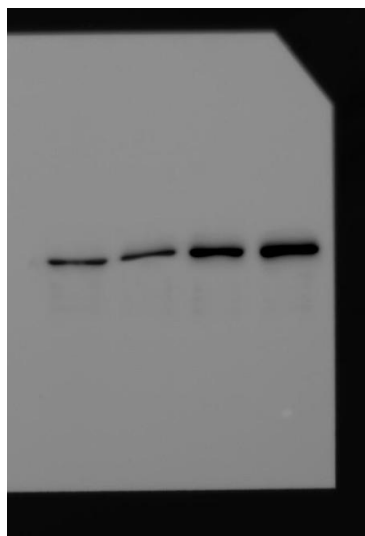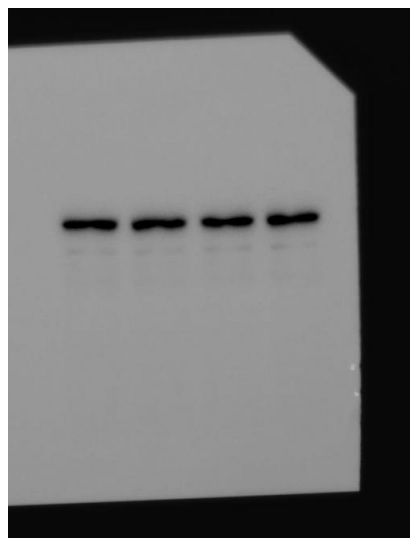

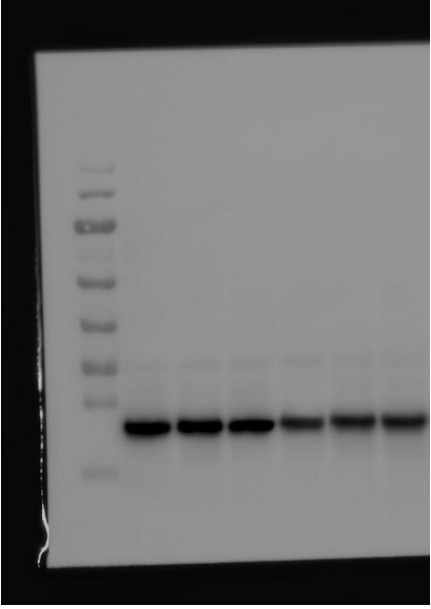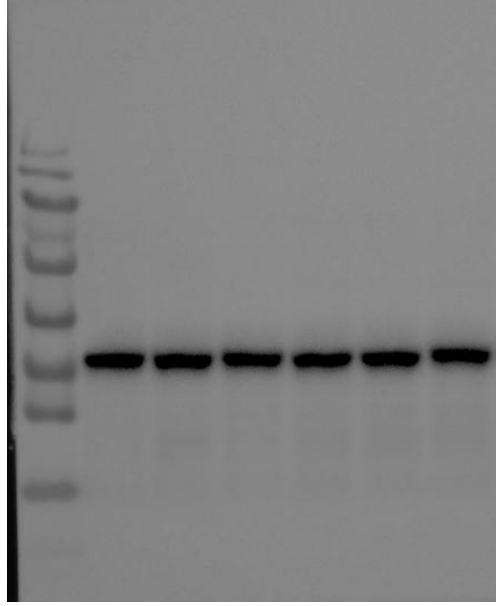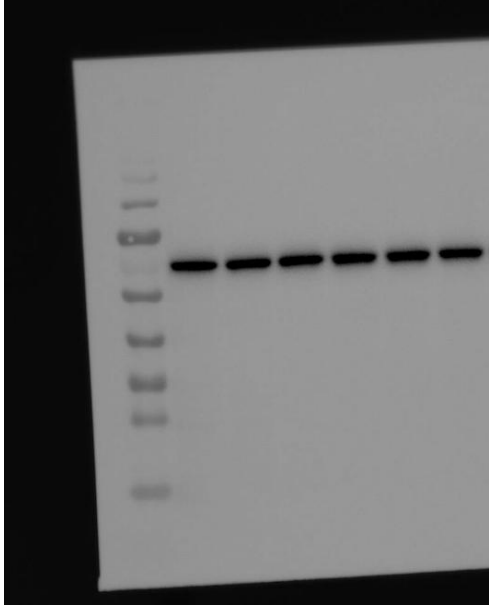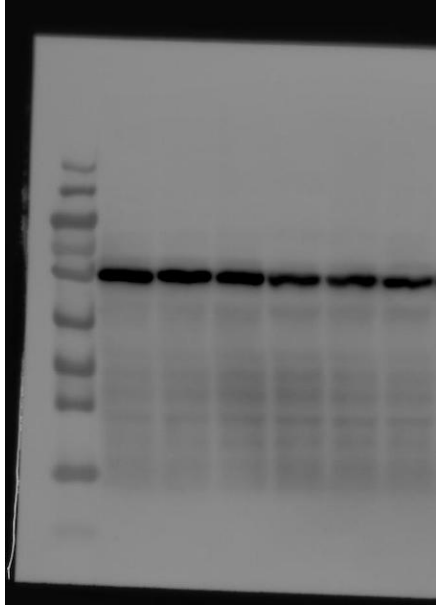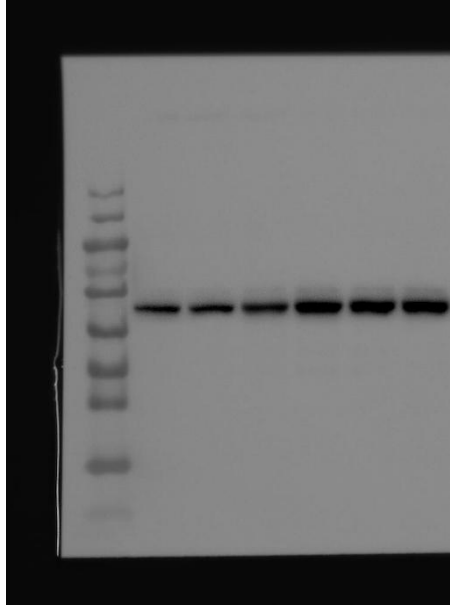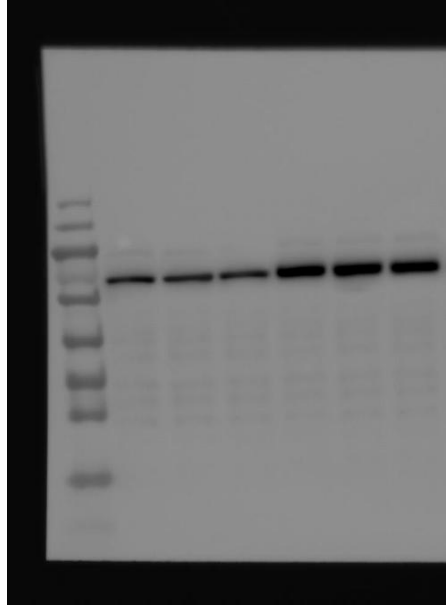

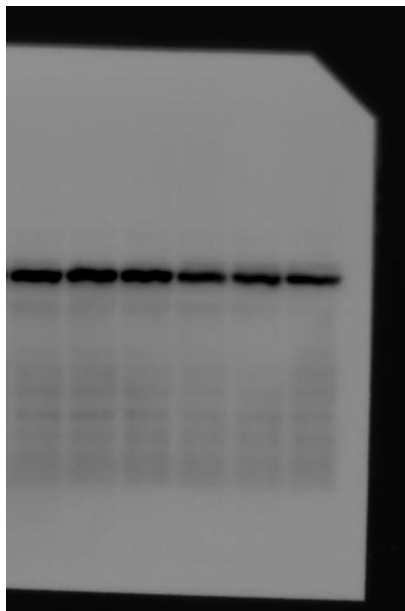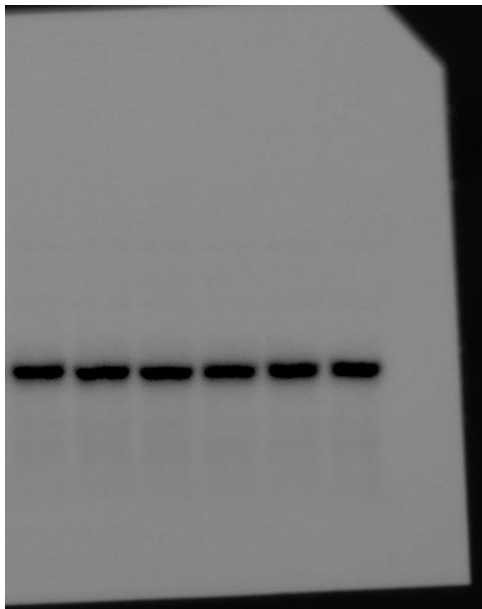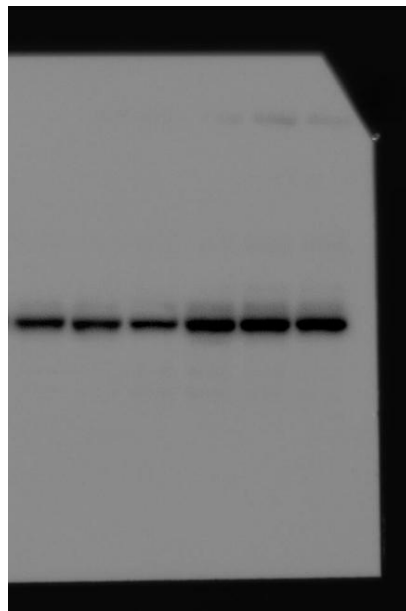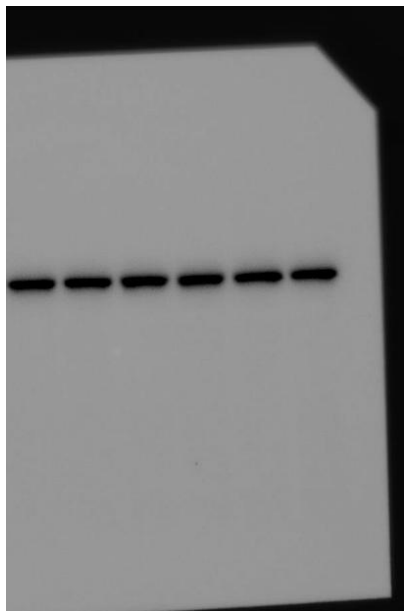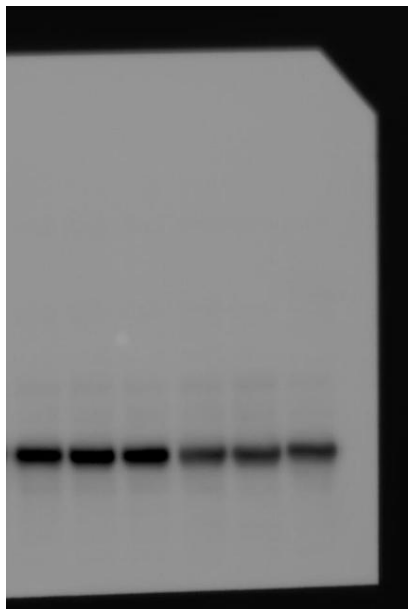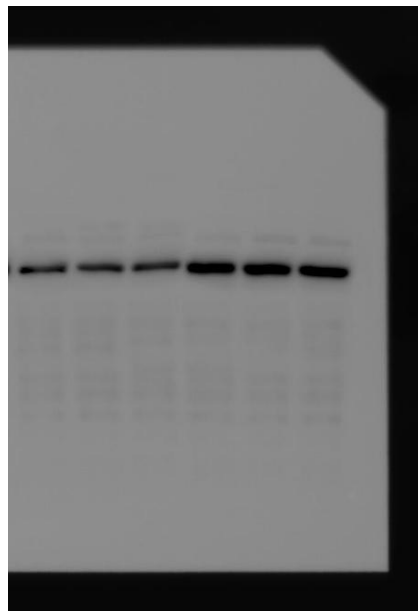

THE  
THE  
THE  
THE

THE  
THE  
THE  
THE

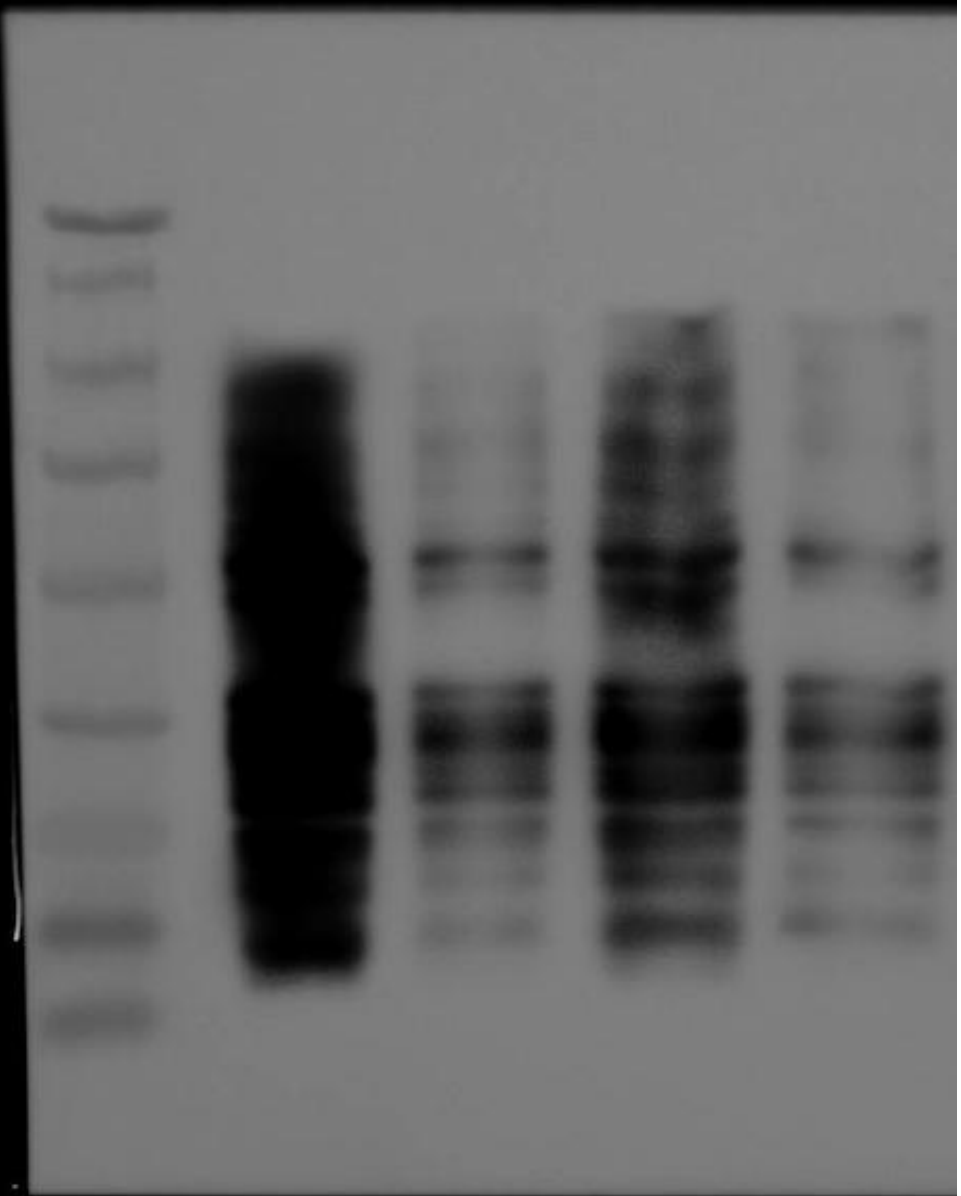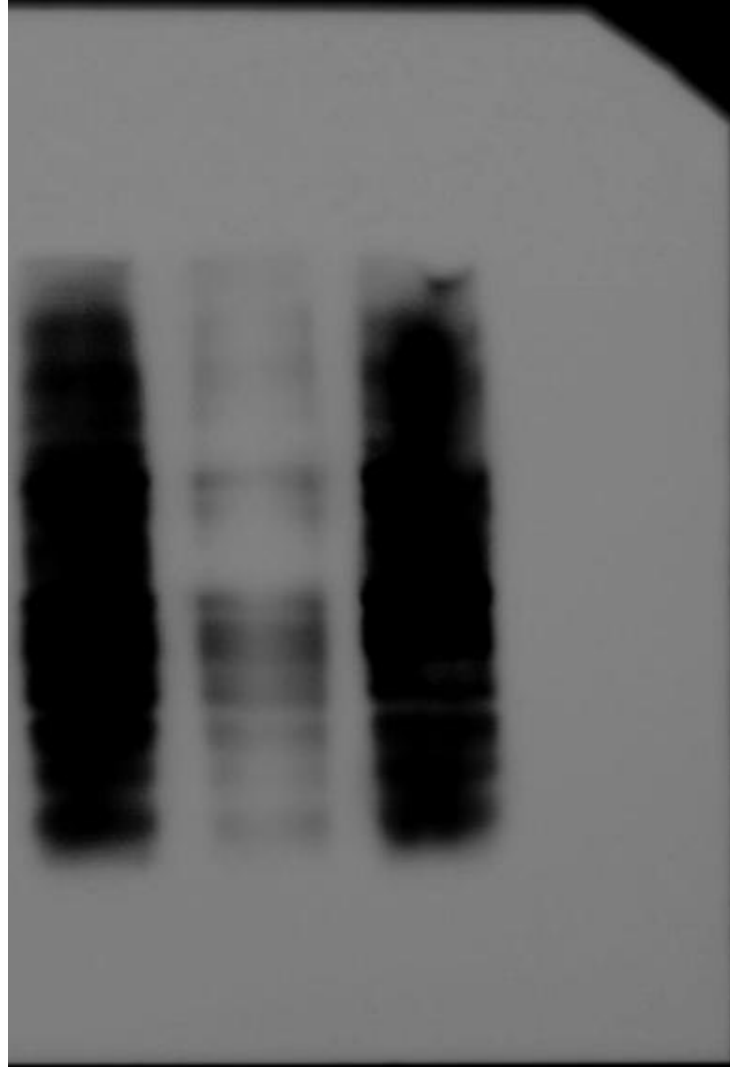

Supplement: Supplementary file 1 — WB [file 41420_2026_3128_MOESM1_ESM.pdf]
