# Supplementary material for: Deubiquitinase USP35 regulates MDM4 degradation to promote endothelial ferroptosis and renal injury progression
Source: Cell Death Discov. 2026 May 25;12:314. doi: 10.1038/s41420-026-03128-5 (PMC13385968; doi:10.1038/s41420-026-03128-5)
Supplement: Supplementary file 2 — Supporting Information [file 41420_2026_3128_MOESM2_ESM.docx]

**Supporting Information**

**Deubiquitinase USP35 Regulates MDM4 Degradation to Promote Endothelial Ferroptosis and Renal Injury Progression**

**
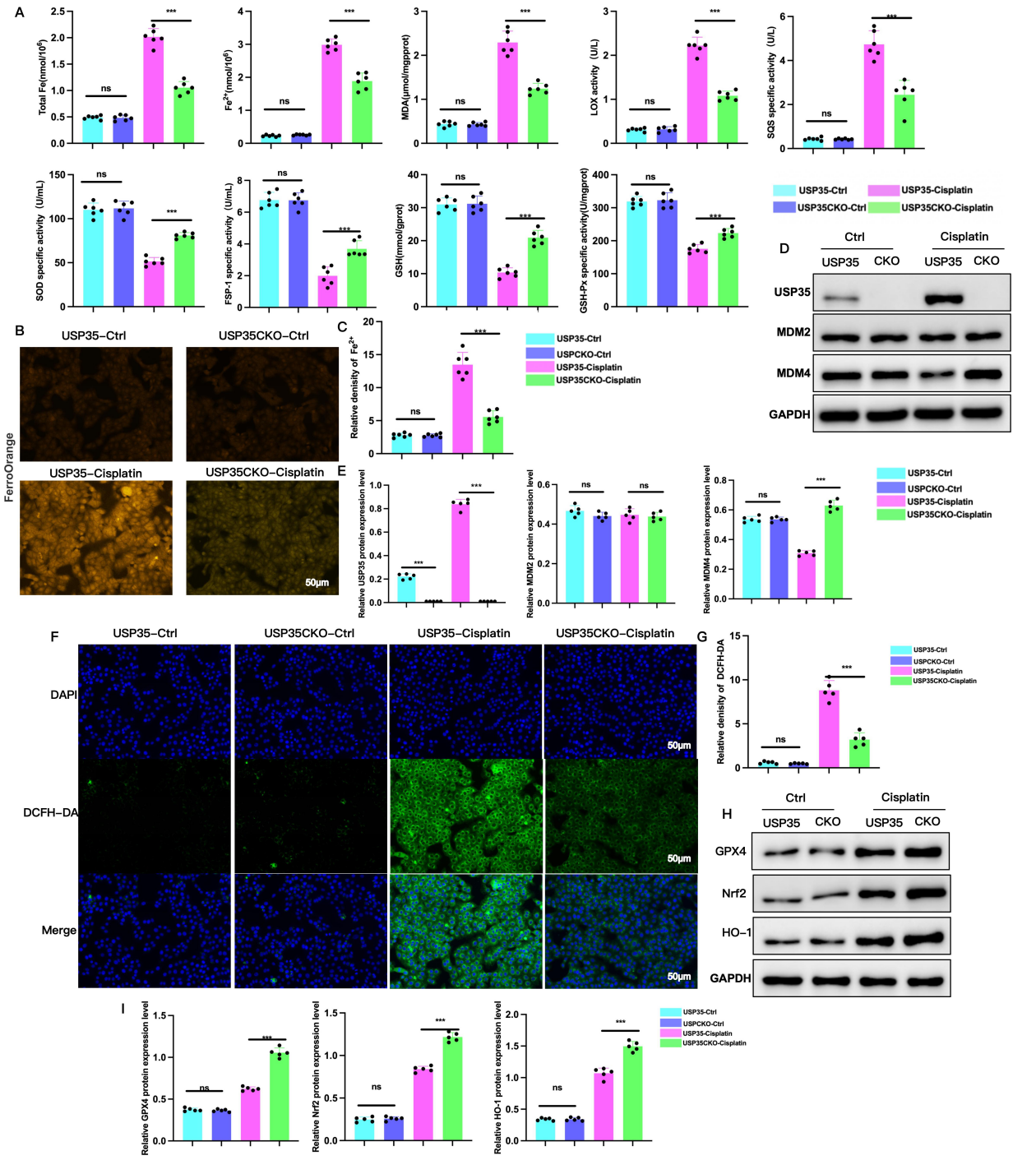
**

**Figure S1: USP35 Conditional Knockout Inhibits Ferroptosis in Renal Endothelial Cells**

A: Detection of ferrous ions, MDA, lipid metabolism indicators (LPO, LOX, SQS), and oxidative damage indicators (SOD, FSP-1, GSH, GSH-Px) in tissues (n=6).

B-C: Staining results and fluorescence intensity analysis of ferrous ions detected by FerroOrange probe (n=6).

D-E: Detection of protein expression levels of USP35, MDM2, and MDM4 (n=6).

F-G: Staining results and fluorescence intensity analysis of ROS detected by DCFH-DA probe (n=6).

H-I: Detection of protein expression levels of ferroptosis-related proteins GPX4, Nrf2, and HO-1 (n=6).

Each point represents an independent experiment. Group comparisons: ^ns^ P>0.05, ^*^P < 0.05, ^**^P<0.01, ^***^P<0.001.


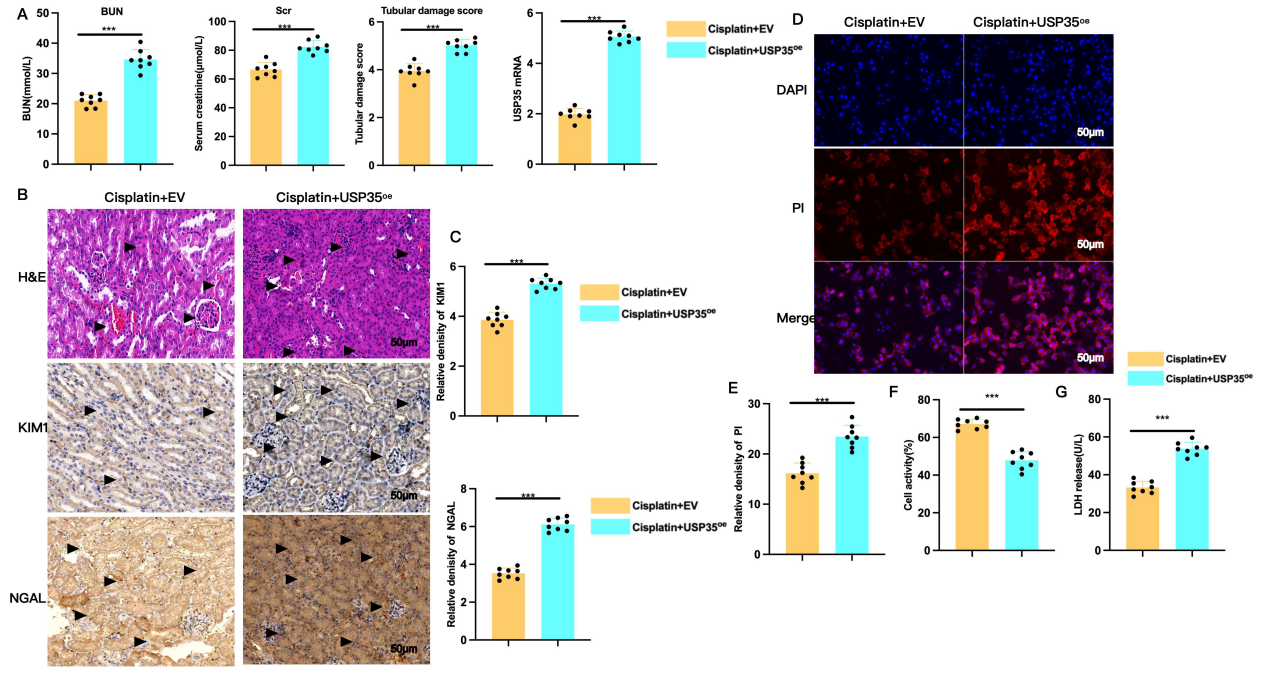


**Figure S2: USP35 Overexpression Promotes Cisplatin-Induced AKI and Endothelial Cell Injury**

A: Renal function test results (n=8).

B-C: H&E staining and staining of renal injury markers (n=8).

D-E: PI staining and fluorescence intensity analysis of endothelial cells (n=8).

F-G: Detection of cell viability and LDH release rate (n=8).

Each point represents an independent experiment. Group comparisons: ^ns^ P>0.05, ^*^P < 0.05, ^**^P<0.01, ^***^P<0.001.

**
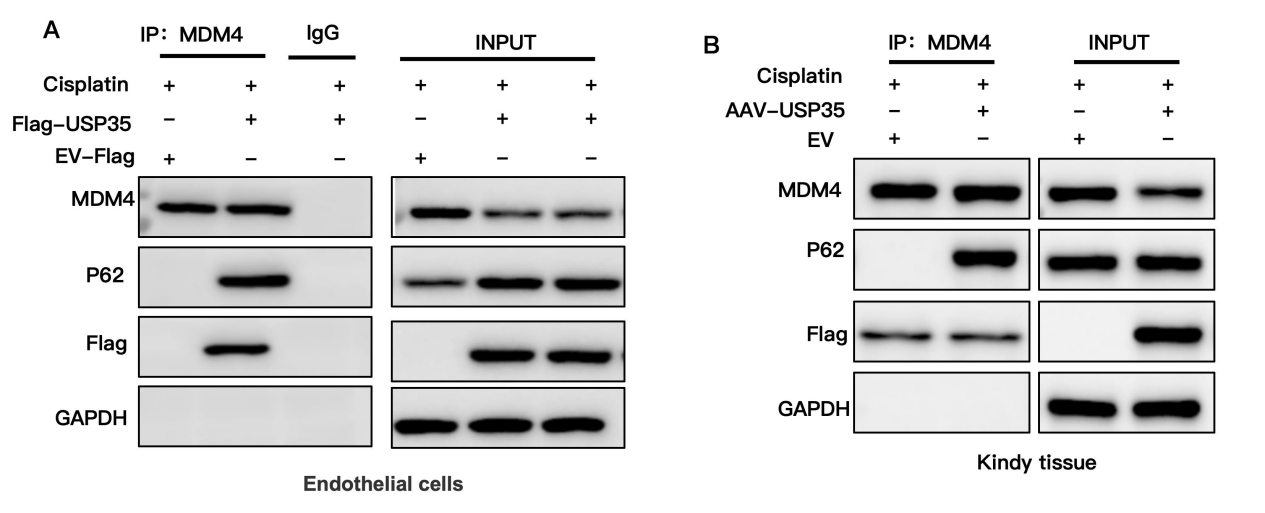
**

**Figure S3: Interaction Between MDM4 and P62**

A: Co-immunoprecipitation of MDM4, P62, and Flag-USP35 in endothelial cells transfected with Flag-USP35 plasmid after cisplatin induction.

B: Co-immunoprecipitation of MDM4, P62, and Flag-USP35 in mouse kidney tissues injected with AAV9--USP35.

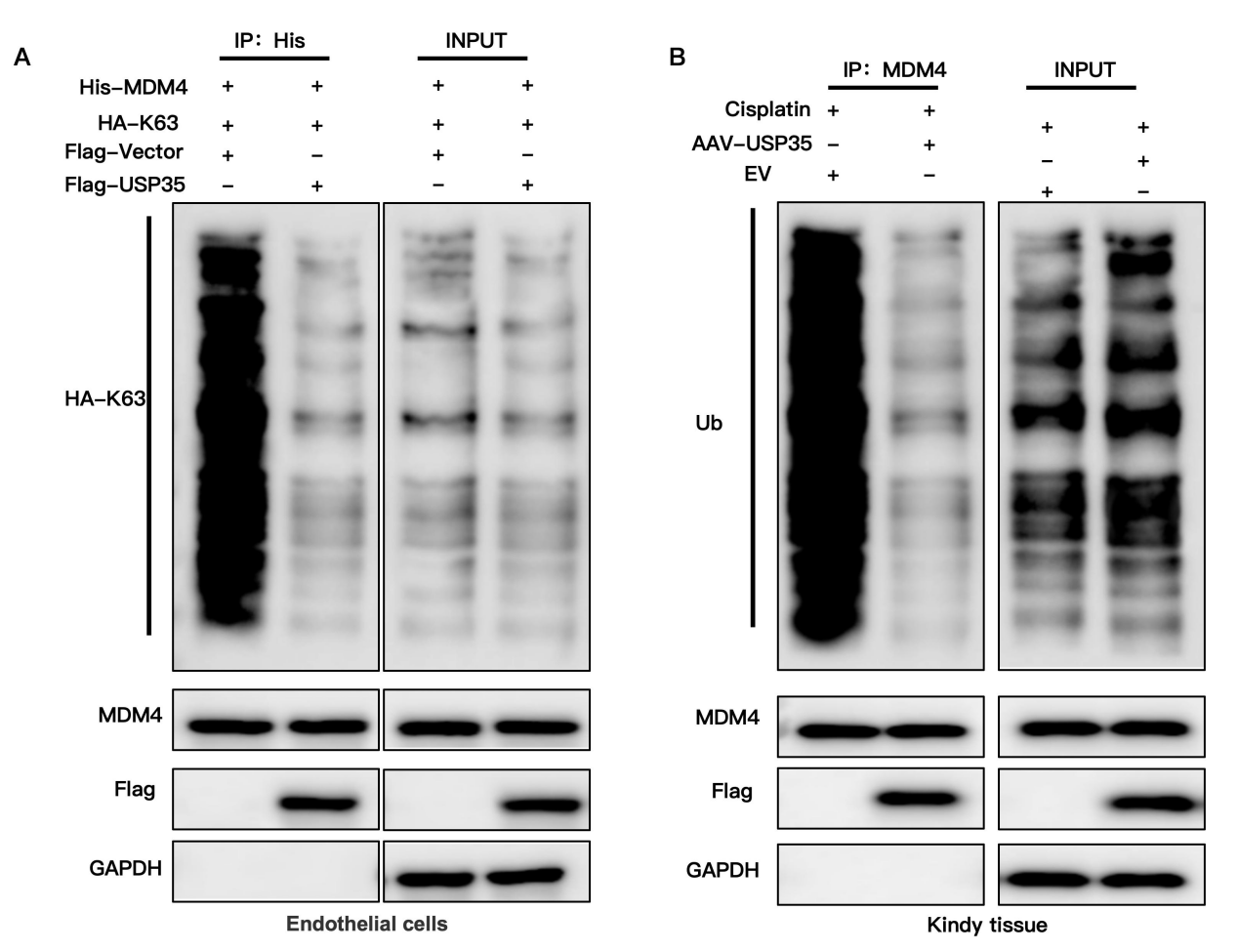


**Figure S4: USP35 Regulates K63 Deubiquitination of MDM4**

A: Cells were co-transfected with His-MDM4, HA-K63, and Flag-USP35, then treated with Baf A1 for 8 hours. IP was performed with anti-His, followed by immunoblotting with antibodies against HA, MDM4, and Flag.

B: Co-IP was performed on kidney tissue lysates from USP35^oe^ and EV mice, followed by immunoblotting with antibodies against UB, Flag, and STING.

**Table S1：Sequences of primers for qPCR used in the study**

| **Gene** | **Species** | **FW** | **RW** |
| --- | --- | --- | --- |
| **USP35** | **Mouse** | **ATCTGTCAGCAACGTCACC** | **CCTTCATCCTCATCCTTGTCTTC** |
| **Actb** | **Mouese** | **CCGTGAAAAGATGACCCAGA** | **TACGACCAGAGGCATACAG** |
|  |  |  |  |
